# QSAR modeling for In vitro assays: linking ToxCast™ database to the integrated modeling framework “OCHEM”

**DOI:** 10.1186/1758-2946-4-S1-P62

**Published:** 2012-05-01

**Authors:** Ahmed Abdelaziz, Iurii Sushko, Wolfram Teetz, Robert Körner, Sergii Novotarskyi, Igor V Tetko

**Affiliations:** 1 Chemoinformatics group, Institute of Bioinformatics & Systems Biology, 85764 Neuherberg, Germany

## 

ToxCast™ project, phases I and II, is testing a combined total of 960 unique chemicals with more than 650 high-throughput assays. The aim of this database is to use advanced science tools to help understand how human body processes are impacted by exposures to chemicals and helps determine which exposures are most likely to lead to adverse health effects.

To better serve this goal and to allow In silico analysis of In vitro assays, we linked the database with an integrated QSAR modeling framework.

The Online Chemical Modeling Environment is a web-based platform that aims to automate and simplify the typical steps required for QSAR modeling. The platform consists of two major subsystems: the database of experimental measurements and the modeling framework. A user-contributed database contains a set of tools for easy input, search and modification of thousands of records. The OCHEM database is based on the wiki principle and focuses primarily on the quality and verifiability of the data. The database is tightly integrated with the modeling framework, which supports all the steps required to create a predictive model: data search, calculation and selection of a vast variety of molecular descriptors, application of machine learning methods, validation, analysis of the model and assessment of the applicability domain. Our intention is to make OCHEM a widely used platform to perform the QSPR/QSAR studies online and share it with other users on the Web.

By such integration, scientists can model In vitro assays using In silico descriptor packages while making benefit of multi-learning features and automatics applicability domain estimation.

http://ochem.eu

